# TRIM59 deficiency promotes M1 macrophage activation and inhibits colorectal cancer through the STAT1 signaling pathway

**DOI:** 10.1038/s41598-024-66388-0

**Published:** 2024-07-12

**Authors:** Haidong Wang, Jun Lou, Hao Liu, Yunlong Liu, Binbin Xie, Wei Zhang, Jiansheng Xie, Hongming Pan, Weidong Han

**Affiliations:** 1 Department of Medical Oncology, Sir Run Run Shaw Hospital, School of Medicine, Zhejiang University, 3# East Qingchun Road, Hangzhou, Zhejiang People’s Republic of China; 2 Laboratory of Cancer Biology, Institute of Clinical Science, Sir Run Run Shaw Hospital, School of Medicine, Zhejiang University, Hangzhou, 310016 Zhejiang People’s Republic of China; 3 Department of Colorectal Medical Oncology, Zhejiang Cancer Hospital, No. 1, East Banshan Road, Gongshu District, Hangzhou, 310022 People’s Republic of China

**Keywords:** TRIM59, Macrophage, Colorectal cancer, M1 polarization, STAT1, Cell signalling, Gastrointestinal cancer

## Abstract

Tumor-associated macrophages play a crucial role in the tumor microenvironment. Tripartite motif 59 (TRIM59), a member of the tripartite motif (TRIM) family, is known to be associated with immunological diseases and macrophage activation. The functional and molecular mechanisms by which TRIM59 affects the occurrence and development of colorectal cancer (CRC) through macrophages are still not well understood. To address this, we generated macrophage-specific TRIM59 conditional knockout mice and utilized these mice to establish colitis-associated cancer and MC38 transplanted CRC models for further investigation. We found that the deficiency of TRIM59 in macrophages inhibited colorectal tumorigenesis in mice. This tumor-suppressive effect was achieved by promoting the activation of M1 macrophages via STAT1 signaling pathway. Further mechanistic studies revealed that TRIM59 could regulate macrophage polarization by ubiquitinating and degrading STAT1. These findings provide evidence that TRIM59 deficiency promotes M1 macrophage activation and inhibits CRC through the STAT1 signaling pathway, suggesting that the TRIM59/STAT1 signaling pathway may be a promising target for CRC.

## Introduction

Colorectal cancer (CRC) is a malignancy with an increasing global incidence^1^. This intricate disease involves multiple pathological factors, mutations, inflammation, gut microbiota, and lifestyle^2^. The development of CRC arises from abnormal differentiation of intestinal epithelial cells^3^. Apart from the tumor itself, the tumor microenvironment (TME) plays a crucial role in therapeutic interventions and patient prognosis^4^. The TME score is a quantitative method to assess TME composition, and is a promising biomarker of prognosis in CRC patients^5^.

Various cellular components comprise the TME, including immune cells, endothelial cells, fibroblast cells, and structural matricellular components^6^. The population of immune cells consists of granulocytes, lymphocytes, and macrophages. Notably, tumor-associated macrophages (TAMs) constitute the majority of immune cells in the TME^7,8^. There are two general types of polarized macrophages: classically activated, pro-inflammatory macrophages (M1), and alternatively activated, pro-repair macrophages (M2)^9^. M1 macrophages are activated by microbial products such as lipopolysaccharide (LPS) or Th1-associated cytokines such as interferon-gamma (IFNγ)^10^. LPS can be recognized by toll-like receptor (TLR) and activate the downstream signaling pathway, including NF-κB and MAPKs^11,12^. IFNγ promotes polarization of M1 macrophages by activation of STAT1 and induces the expression of STAT1-responsive genes, such as CXCL9, CXCL10, and CXCL11^13,14^. The combination of LPS and IFNγ can further enhance the activation of M1 macrophages and their secretion of cytokines^15–17^. M1 macrophages produce pro-inflammatory cytokines, including nitric oxide synthase (iNOS), tumor necrosis factor (TNF) α, IL-1β, and IL-6, etc., which play an inhibitory role in tumorigenesis^13,18^. In contrast, IL-4 or IL-13 activates M2 macrophages, which promote angiogenesis and tumor growth^19^.

Generally, TAMs are recognized as M2 macrophages within tumors, actively promoting tumor growth and metastasis^20,21^. However, TAMs are highly adaptable and can be induced to polarize into M1 macrophages, which exhibit anti-tumor activity^22^. Current strategies for cancer treatment involving macrophages include macrophage depletion, manipulation of macrophage recruitment, and repolarization of macrophages^23^. In the early stages, a low dose of diphenyleneiodonium (DPI) inhibits M1 macrophages polarization and subsequently inhibits CAC^24^. Class IIa histone deacetylase (HDAC) inhibitors can reprogram TAMs in breast cancer, leading to an anti-tumor effect and improved efficacy of chemotherapy and immunotherapy^25^. Repolarization of TAMs into tumoricidal macrophages has been observed with the use of MPLA and IFNγ^26^. Furthermore, genetic changes can influence macrophage polarization. For example, the deficiency of PDCD4 enhances macrophage anti-tumor activity by increasing TFEB expression^27^. The deficiency of xCT has been shown to limit tumorigenicity and metastasis in a mouse model of hepatocellular carcinoma (HCC) by reducing TAMs recruitment and infiltration, as well as inhibiting M2 polarization^28^. PKN2 inhibits M2 polarization and tumor growth in colon cancer cells^29^. However, the mechanisms underlying macrophage polarization in the TME remain complex and unclear. Therefore, the control of macrophage polarization in the TME requires further investigation.

The tripartite motif (TRIM) family proteins function as E3 ubiquitin ligases and possess a distinctive structural composition comprising a RING-finger domain, one or two zinc-finger domains (B-Box), and its associated coiled-coiled domain. TRIM proteins exhibit diverse biological roles, including immunity, carcinogenesis, autophagy, and antiviral activity^30,31^. TRIM59, a member of the TRIM family, possesses a RING-finger domain, a B-Box, an associated coiled-coiled region, and a transmembrane region (TM)^30^. Being upregulated in a wide variety of cancerous tissues, including lung, breast, gastric, liver, colon, and renal carcinomas, TRIM59 may serve as an innovative diagnostic and prognostic marker^32–38^. TRIM59 plays a role in promoting the proliferation of CRC and facilitating metastasis through the PI3K/AKT signaling pathway^39^. Additionally, TRIM59 is implicated in BCG-activated macrophage cytotoxicity^40^, and exhibits protective effects against sepsis in macrophages^41^. However, the exact role of TRIM59 in macrophages for the development of CRC remains unclear.

In our study, it was observed that the deficiency of TRIM59 in macrophages inhibited the CAC and promoted M1 macrophages polarization. Moreover, in vitro experiments revealed that TRIM59 deficiency in macrophages promoted M1 macrophages polarization through the STAT1 signaling pathway. We further elucidated the mechanism underlying this phenomenon, which involved the interaction between TRIM59 and STAT1, leading to the ubiquitination and degradation of STAT1. Additionally, in the MC38 transplanted tumor model, the knockout of TRIM59 resulted in increased activation of M1 macrophages. Based on these findings, we can conclude that TRIM59 deficiency promotes M1 macrophage activation and inhibits CRC via the STAT1 signaling pathway.

## Materials and methods

### Animals

Using TRIM59 flox/flox micewith floxed alleles (Gempharmatech Co., Ltd, Nanjing, China), we crossed with Lyz-cre or Villin-cre mice (Gempharmatech Co., Ltd, Nanjing, China) to generate TRIM59 conditional knockout mice. For all experiments, TRIM59^f/f^ mice without Cre transgene were used as control mice. All institutional and national guidelines for the care and use of laboratory animals were followed and were approved by the Medical Ethics Committee of Sir Run Run Shaw Hospital, School of Medicine, Zhejiang University (SRRSH202302100).

### Animal models

#### Subcutaneous transplanted tumor model

8–10-weeks-old TRIM59^f/f^ and TRIM59^f/f^ Lyz2-cre female mice were subcutaneously injected with 1 × 10^6^ cells of MC38 colon carcinoma cells into their backs. Approximately 14 days after subcutaneous injection, tumors were harvested from the mice.

#### Induction of colitis and colitis-associated colorectal cancer

8–10-weeks-old TRIM59^f/f^ and TRIM59^f/f^ Lyz2-cre female mice were administered DSS (2% in drinking water) (0216011010, MP Biomedicals, MW: 36,000–50,000 Da, USA) for 7 days, followed by normal drinking water for another 7 days to induce colitis mice model.

8–10-weeks-old TRIM59^f/f^ and TRIM59^f/f^ Lyz2-cre as well as TRIM59^f/f^ and TRIM59^f/f^ Villin-cre female mice were intraperitoneally injected with 10 mg/kg azoxymethane (AOM) (A5486, Sigma, USA). One week later, the mice were given DSS (2% in drinking water) for 1 week, followed by normal drinking water for 2 weeks to induce the colitis-associated cancer mice model. This cycle was repeated twice before sacrificing the mice.

### Cell culture and treatment

For the bone marrow-derived macrophage (BMDM) culture, bone marrow were obtained from the femurs and tibias of mice. The bone marrow cells were cultured in Dulbecco's Modified Eagle Medium (DMEM) (FD7144, Fudebio, Hangzhou, China) with 10% fetal bovine serum (FBS) (NFBS-2500A, Noverse, South America) and 100 ng/mL M-CSF (315-02, Peprotech, USA) for 6 days.

HEK293T cells, MC38 cells, and RAW264.7 cells were purchased from the Cell Bank of the Chinese Academy of Science and maintained in DMEM with 10% FBS at 37 °C and 5% CO_2_.

BMDMs and RAW264.7 cells were treated with LPS (L2880, Sigma, USA) at 500 ng/mL, or IFNγ (RP01070, Abclonal Biotechnology, China) at 40 ng/mL for different time periods.

### Plasmid construction

The *TRIM59* gene was fused with the Flag or Myc tag and inserted into pcDNA3.1(+) or pLVX-Puro vector. The *STAT1* gene was fused with the Flag or Myc tag and inserted into pcDNA3.1(+) vector. HA-Ub, HA-Ub-K48O and HA-Ub-K63O were from Miao Ling Biotechnology Co., Ltd (Wuhan, China).

### Co-immunoprecipitation (Co-IP)

For co-immunoprecipitation, whole cell extracts were lysed in the NP-40 lysis buffer and then centrifuged at 14,000×*g* for 10 min. A total of 10% of lysates were taken as input, and the remaining lysates were used for IP. The remaining 90% of the lysates were pre-incubated and rotated with an anti-Myc antibody (2276, CST) overnight at 4 °C. Protein G beads (17061802, Cytiva, USA) were added to the lysates and rotated for 4 h at 4 °C. The beads were then washed three times with lysis buffer after precipitation. The NP-40 lysis buffer contains 1% NP-40, 50 mM Tris–HCl (pH 7.4) and 150 mM NaCl.

For mass spectrometry experiments, HEK293T cells overexpressing Myc-tagged TRIM59 were harvested and lysed at 4 °C. Then the IP experiment was performed as described above. The sample was sent to BGI (BGI, Shenzhen, China) for mass spectrometry and further analysis.

### Immunofluorescence staining

The samples were fixed with 4% paraformaldehyde, permeabilized with 0.5% Triton X-100, and then blocked with 5% BSA for 1 h. Subsequently, they were incubated overnight at 4 °C with antibodies against Myc (2276, CST) and STAT1 (14994, CST). After that, the samples were incubated with secondary antibodies, DyLight 488, Goat Anti-Mouse IgG (A23210, Abbkine, China) and Dylight 594, Goat Anti-Rabbit IgG (A23420, Abbkine, China). The nuclei were stained with 4′,6-diamidino-2-phenylindole (DAPI). The images were obtained using a Zeiss LSM 710 confocal microscope system (Carl Zeiss, Germany).

### RNA isolation and quantitative real-time PCR (QPCR) analysis

Total RNA was extracted from cells and tissues using Total RNA Extraction Reagent (R401-01-AA, Vazyme, China). The cDNA was synthesized using Accurate Biotechnology Reverse Transcription Kit (AG11728, ACCURATE BIOTECHNOLOGY, China), and Quantitative real-time PCR was performed using SYBR Green Pro Taq HS Premix qPCR Kit (AG11701, ACCURATE BIOTECHNOLOGY, China). The primer sequences are listed in Supplementary Table S1.

### Western blotting analysis

After treatment with LPS or IFNγ, the total protein from the BMDMs or RAW264.7 cells was collected using NP-40 lysis buffer (BL653A, Biosharp, China) supplemented with a protease inhibitor cocktail (B13001, Selleck, China). The different tissues were collected, then they were intercepted and homogenized in NP-40 lysis buffer with a protease inhibitor cocktail together with grinding beads. The proteins were separated by 10% SDS-PAGE and then transferred to a PVDF membrane (1620177, Bio-Rad, CA), and incubated with the primary antibody listed in Supplementary Table S2 overnight at 4 °C. The signals were visualized using enhanced chemiluminescence (Amersham Imager 600, GE, MA).

### Flow cytometry

The tumors were minced and digested in RPMI-1640 media containing 2 mg/mL collagenase IV (Biosharp, China) and 2% FBS for 1 h at 37 °C. After filtering, the cells were centrifuged at 400×*g* for 10 min and resuspended in PBS. The cells were then stained with APC anti-CD45 (clone 30-F11, BioLegend, CA), BV421 anti-mouse/human CD11b (clone M1/70, BioLegend, CA), PE anti-mouse F4/80 (clone BM8, BioLegend, CA), APC/Cyanine7 anti-mouse MHCII (clone M5/114.15.2, BioLegend, CA). The data were analyzed by FlowJo software (Ashland, OR). The macrophage analysis process is illustrated in Supplementary Fig. S1.

### Cytokine determination

The levels of IL-1β, IL-6, MCP-1, IL-12 p40, and TNF-α in the supernatant or serum were analyzed using ELISA kits (Invitrogen, USA) following the manufacturer’s instructions.

### Statistical analysis

The data are presented as means ± standard error of the mean (SEM). Statistical analysis was performed using GraphPad Prism software (GraphPad, San Diego, CA). Differences between groups were assessed using the Student’s t test and one-way ANOVA. Statistical significance was denoted at ***p < 0.001, **p < 0.01, and *p < 0.05.

## Results

### TRIM59 deficiency in macrophages, but not in intestinal epithelium, inhibits the development of CAC

It is well-established that gene expression is closely linked to its functional role. To investigate the function of TRIM59, we initially assessed its expression in various mouse tissues. Through the utilization of Quantitative polymerase chain reaction (QPCR) and western blotting (WB), we observed a significantly higher expression of TRIM59 in immune-related organs, such as the spleen, thymus, and lymph nodes (Supplementary Fig. S2). The results indicate that TRIM59 is involved in immune diseases such as infection, inflammation, and tumor^37^.

To investigate the role of TRIM59 in colitis and cancer, we generated TRIM59^f/f^ Lyz2-cre mice with a conditional knockout of TRIM59 specifically in macrophages (Supplementary Fig. S3A). We also compared them to TRIM59^f/f^ Villin-cre mice with a conditional knockout of TRIM59 in intestinal epithelium. The level of TRIM59 in BMDMs was assessed using QPCR and WB, which confirmed a significant reduction in TRIM59 expression in macrophages (Supplementary Fig. S3B,C). Flow cytometry was utilized to examine the effects of TRIM59 knockout on macrophages, revealing no observable differences in the total of cells and macrophage percentage after TRIM59 knockout (Supplementary Fig. S3D). Additionally, the surface markers of macrophages remained unchanged (Supplementary Fig. S3E). These findings suggest that TRIM59 deficiency in macrophages does not affect macrophage maturation.

**Figure 1:**
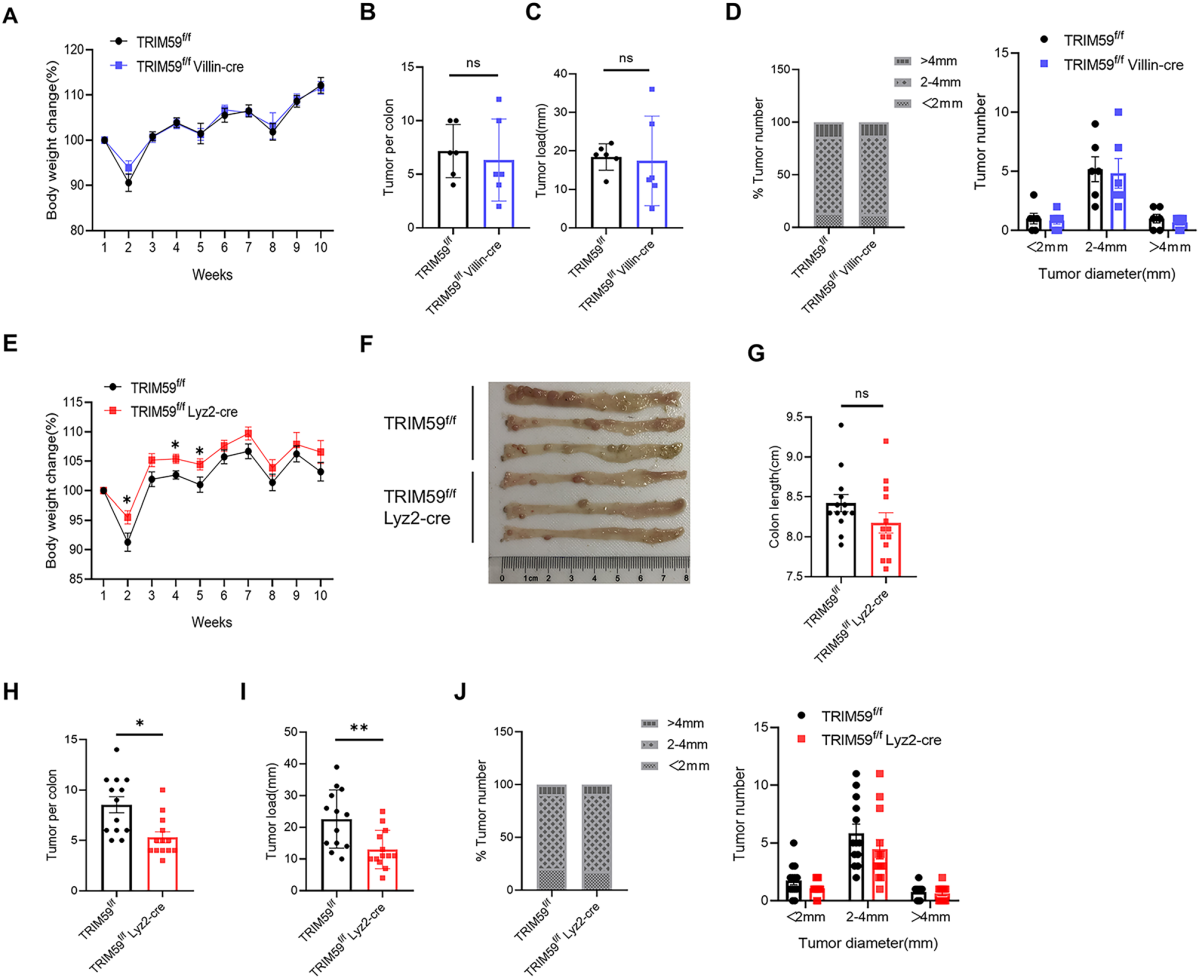
TRIM59 deficiency in macrophages, but not in intestinal epithelium, inhibits the development of CAC. (**A**–**D**) Percentage change in body weight (**A**), tumor number (**B**), tumor load (**C**), and tumor distribution with classified tumor size (**D**) in TRIM59^f/f^ and TRIM59^f/f^ Villin-cre mice (n = 7–8 per group). (**E**–**J**) Percentage change in body weight (**E**), representative image of CAC tumors (**F**), colon length (**G**), tumor number (**H**), tumor load (**I**), and tumor distribution with classified tumor size (**J**) in TRIM59^f/f^ and TRIM59^f/f^ Lyz2-cre mice (n = 15–16 per group). The data are represented as mean ± SEM, *p < 0.05, **p < 0.01, ***p < 0.001.

To explore the role of TRIM59 in colon carcinogenesis, we utilized an AOM/DSS-induced CAC mice model. The percentage of body weight was monitored weekly, and after three cycles of DSS treatment, the mice were sacrificed for tumor analysis. In the TRIM59^f/f^ Villin-cre mice, no significant differences were observed in terms of body weight change (Fig. 1A), tumor number (Fig. 1B), tumor load (Fig. 1C), tumor size and size distribution (Fig. 1D) compared to the TRIM59^f/f^ mice. However, the TRIM59^f/f^ Lyz2-cre mice exhibited greater tolerance to DSS treatment compared to the TRIM59^f/f^ mice (Fig. 1E), although there was no difference observed in colon length (Fig. 1F,G). Notably, it was observed that the TRIM59^f/f^ Lyz2-cre mice had a lower tumor number (Fig. 1H) and tumor load (Fig. 1I) compared to the TRIM59^f/f^ mice, but no difference was observed in the size distribution of tumors (Fig. 1J). These results clearly demonstrate that the deficiency of TRIM59 in macrophages, rather than in intestinal epithelium,effectively inhibits the development of CAC.

### TRIM59 deficiency in macrophages inhibits CAC by promoting M1 macrophages polarization

**Figure 2:**
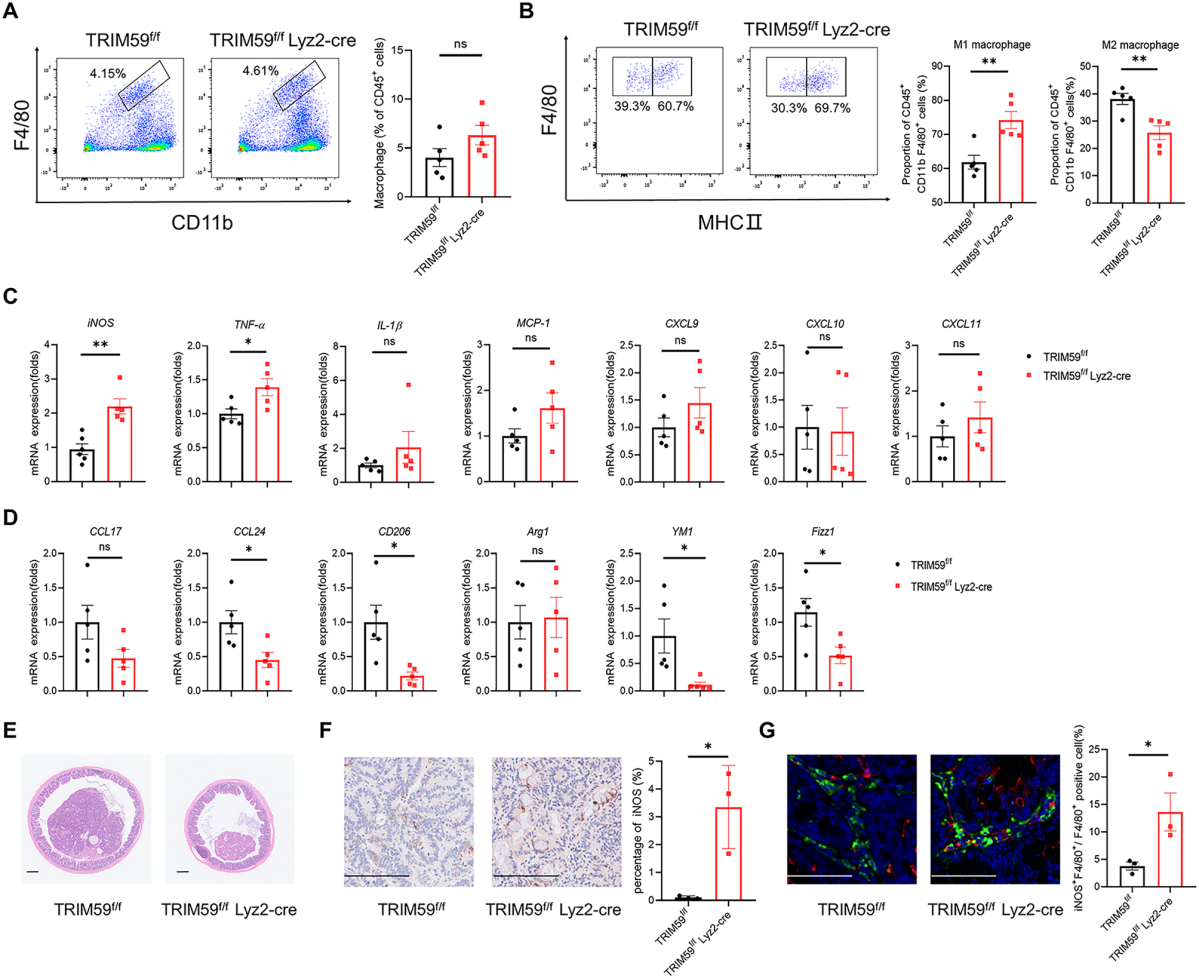
TRIM59 deficiency in macrophages inhibits CAC by promoting M1 macrophages polarization. (**A**,**B**) Flow cytometry analysis of macrophage proportion (**A**) and the proportion of M1 and M2 macrophages (**B**) (n = 5 per group). (**C**) QPCR analysis of chemokines and pro-inflammatory cytokines in CAC tumors (n = 5 per group). (**D**) QPCR analysis of M2 macrophage markers in MC38 tumors (n = 5 per group). (**E**) Representative H&E staining of CAC tumors. (Scale bar = 200 μm). (**F**) IHC staining for iNOS in CAC tumors (Scale bars = 100 μm) and quantitative analysis of iNOS positive area (%) (n = 3 per group). (**G**) Representative immunofluorescence staining of F4/80 (green) and iNOS (red) in CAC tumors (Scale bars = 100 μm) and quantitative analysis of iNOS^+^ F4/80^+^ cells (n = 3 per group). The data are represented as mean ± SEM, *p < 0.05, **p < 0.01, ***p < 0.001.

Macrophage plays a crucial role in inflammatory response and tumor immunity within the colon^42^. The expression of MHCII has been demonstrated to distinguish TAMs into distinct subpopulations. MHCII^hi^ TAMs resemble M1-like macrophages, whereas MHCII^lo^ TAMs resemble M2-like macrophages^43^. To investigate changes in macrophage populations after AOM/DSS treatment, the proportion of macrophages within CAC tumors in mice was analyzed using flow cytometry. The percentage of macrophages showed no significant change (Fig. 2A). However, in TRIM59^f/f^ Lyz2-cre mice, the proportion of M1 macrophages was upregulated, while the proportion of M2 macrophages was downregulated (Fig. 2B). QPCR analysis was performed to further examine the expression of pro-inflammatory cytokines and chemokines. The level of *iNOS* and *TNF-α* were elevated in TRIM59^f/f^ Lyz2-cre mice. Although there were no statistically significant differences in the levels of *MCP-1*, *IL-1β*, *CXCL9*, *CXCL10*, and *CXCL11*, an increasing trend was observed in TRIM59^f/f^ Lyz2-cre mice (Fig. 2C). Also, the markers of M2 macrophage (*CCL24*, *CD206*, *YM1* and *Fizz1*) were downregulated in TRIM59^f/f^ Lyz2-cre mice (Fig. 2D). H&E staining showed the presence of tumors in CAC (Fig. 2E). IHC and immunofluorescence were performed to examine the level of iNOS in CAC tumors, which is an M1 macrophage marker. It was found that the iNOS positive area (%) was higher in TRIM59^f/f^ Lyz2-cre mice (Fig. 2F). Accordingly, the TRIM59^f/f^ Lyz2-cre mice had an increased ratio of iNOS^+^ F4/80^+^ cells than TRIM59^f/f^ mice (Fig. 2G). The results showed higher activation of M1 macrophages in TRIM59^f/f^ Lyz2-cre mice. Taken together, these findings provide support for the notion that TRIM59 deficiency in macrophages inhibits CAC by promoting polarization towards M1 macrophages.

The transition from colitis to CAC is a slow and continuous process^44^. We established an acute colitis mice model to investigate this transition. The assessment of body weight change (Supplementary Fig. S4A), survival curve (Supplementary Fig. S4B), disease activity index (Supplementary Fig. S4C), and colon length (Supplementary Fig. S4D) revealed no significant changes. Furthermore, the QPCR analysis of pro-inflammatory cytokines in the colorectum also showed no significant differences (Supplementary Fig. S4E). These results indicate that TRIM59 deficiency in macrophages does not have an impact on DSS-induced colitis.

### TRIM59 deficiency in macrophages promotes M1 macrophages polarization through the STAT1 signaling pathway

M1 macrophages can be activated by LPS or IFNγ^45^. To investigate the function of TRIM59 in macrophages, we stimulated BMDMs with 500 ng/mL LPS or 40 ng/mL IFNγ at different time points. Upon LPS treatment, no significant differences were observed between the two groups. The levels of pro-inflammatory cytokines, including *IL-1β*, *IL-6*, *iNOS*, *MCP-1*, *IL-12 p40*, and *TNF-α*, remained unchanged (Supplementary Fig. S5A). Additionally, there were no changes detected in the expression of MAPK and NF-κB signaling pathways (Supplementary Fig. S5B). The levels of pro-inflammatory cytokines IL-6, TNF-α, and IL-12 p40 in the supernatant of LPS stimulation BMDMs also showed no significant difference when measured using ELISA (Supplementary Fig. S5C). To further investigate the in vivo response, we induced a sepsis mice model by injecting 15 mg/kg LPS. As expected, there was no difference in the survival rate between the two groups (Supplementary Fig. S5D). The concentrations of pro-inflammatory cytokines, including IL-1β, IL-6, MCP-1, and TNF-α, in the serum of sepsis mice were measured using ELISA, and no differences were observed (Supplementary Fig. S5E). These results indicate that TRIM59 deficiency in macrophages does not affect LPS-induced sepsis in mice.

**Figure 3:**
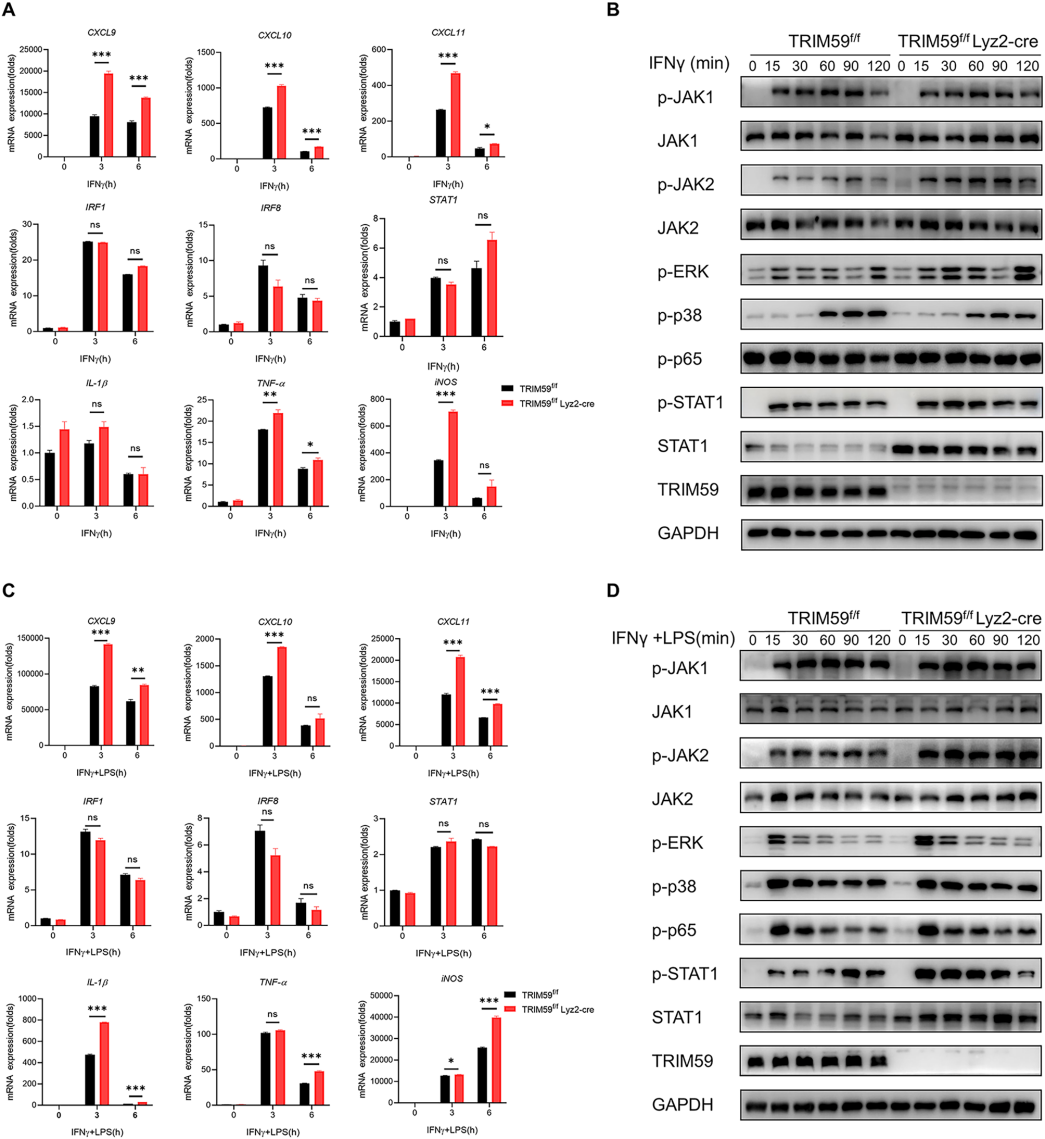
TRIM59 deficiency in macrophages promotes M1 macrophages polarization via STAT1 signaling pathway. (**A**) QPCR analysis of chemokines and pro-inflammatory cytokines in BMDMs treated with IFNγ (40 ng/mL) for 3 and 6 h (n = 3 per group). (**B**) WB analysis of JAK/STAT1, NF-κB, and MAPK signaling pathways in BMDMs treated with IFNγ (40 ng/mL) for 15, 30, 60, 90 and 120 min. (**C**) QPCR analysis of chemokines and pro-inflammatory cytokines in BMDMs treated with IFNγ (40 ng/mL) and LPS (500 ng/mL) for 3 and 6 h (n = 3 per group). (**D**) WB analysis of of JAK/STAT1, NF-κB, and MAPK signaling pathways in BMDMs treated with IFNγ (40 ng/mL) and LPS (500 ng/mL) for 15, 30, 60, 90 and 120 min. The experiments were repeated three times. The data are represented as mean ± SEM, *p < 0.05, **p < 0.01, ***p < 0.001.

Furthermore, we conducted experiments to stimulate BMDMs with IFNγ. Interestingly, we observed an upregulation of the chemokines *CXCL9*, *CXCL10*, and *CXCL11* in TRIM59-knockout BMDMs treated with IFNγ at 3 and 6 h. Additionally, the pro-inflammatory cytokines *TNF-α* and *iNOS* were also found to be increased in TRIM59-knockout BMDMs. The mRNA levels of *STAT1* and its downstream genes *IRF1* and *IRF8* were not significantly different between groups (Fig. 3A). Further mechanistic study revealed that upon treatment of TRIM59-knockout BMDMs with IFNγ, a significant increase in STAT1 expression. Meanwhile, the level of phosphorylated STAT1 was increased in TRIM59-knockout BMDMs treated with IFNγ at 30 min. However, key proteins in the JAKs, MAPK, and NF-κB signaling pathway showed no significant changes (Fig. 3B). Additionally, we used IFNγ and LPS as co-stimulants for BMDMs, and the outcomes obtained were comparable to those observed with IFNγ treatment alone (Fig. 3C,D). These data confirm that TRIM59 deficiency in macrophages promotes M1 macrophages polarization through the STAT1 signaling pathway.

**Figure 4:**
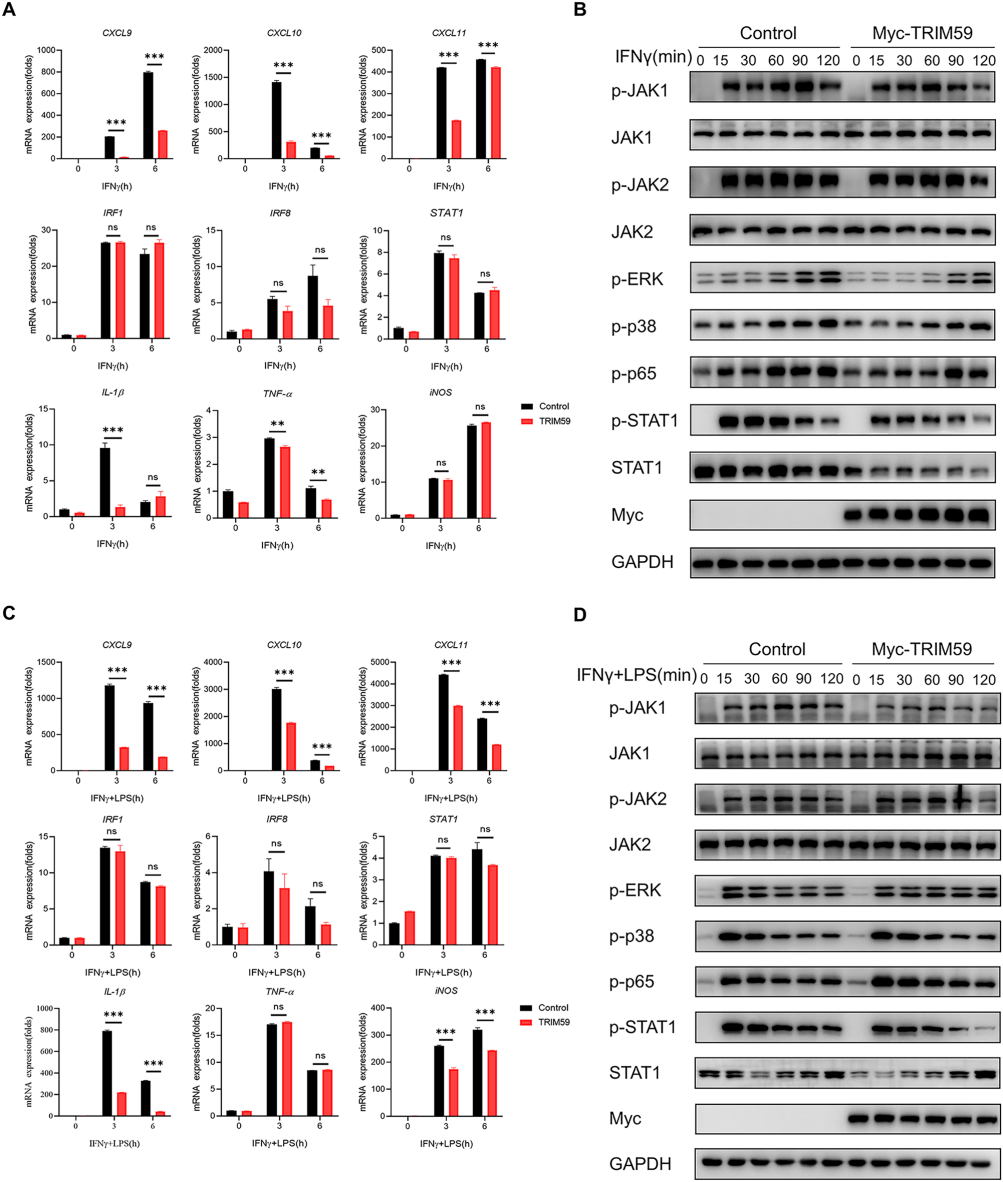
TRIM59 overexpression inhibits M1 macrophage activation. (**A**) QPCR analysis of chemokines and pro-inflammatory cytokines in RAW264.7 cells treated with IFNγ (40 ng/mL) for 3 and 6 h (n = 3 per group). (**B**) WB analysis of JAK/STAT1, NF-κB, and MAPK signaling pathways in RAW264.7 cells treated with IFNγ (40 ng/mL) for 15, 30, 60, 90 and 120 min. (**C**) QPCR analysis of chemokines and pro-inflammatory cytokines in RAW264.7 cells treated with IFNγ (40 ng/mL) and LPS (500 ng/mL) for 3 and 6 h (n = 3 per group). (**D**) WB analysis of JAK/STAT1, NF-κB, and MAPK signaling pathways in RAW264.7 cells treated with IFNγ (40 ng/mL) and LPS (500 ng/mL) for 15, 30, 60, 90 and 120 min. The experiments were repeated three times. The data are represented as mean ± SEM, **p < 0.01, ***p < 0.001.

To further investigate the role and mechanism of TRIM59 in macrophages, we established stable RAW264.7 cells line over-expressing TRIM59. The same approach was used to confirm the function of TRIM59 on macrophage activation, as observed in BMDMs. Consistent with our expectation, the chemokines *CXCL9*, *CXCL10*, and *CXCL11* were downregulated in TRIM59 over-expressing cells treated with IFNγ (Fig. 4A). Moreover, we observed a reduction in the levels of STAT1 in TRIM59-overexpression cells. The phosphorylated STAT1 was also reduced in TRIM59-overexpression cells treated with IFNγ at 15 and 30 min. There were no significant changes in the proteins associated with JAKs, the MAPK, and NF-κB signaling pathway (Fig. 4B). Co-stimulation with IFNγ and LPS yielded similar results to the IFNγ treatment alone (Fig. 4C,D). These results indicate that TRIM59 inhibits M1 macrophage activation, consistent with the results obtained in TRIM59 knockout BMDMs.

### TRIM59 ubiquitinates and degrades STAT1

**Figure 5:**
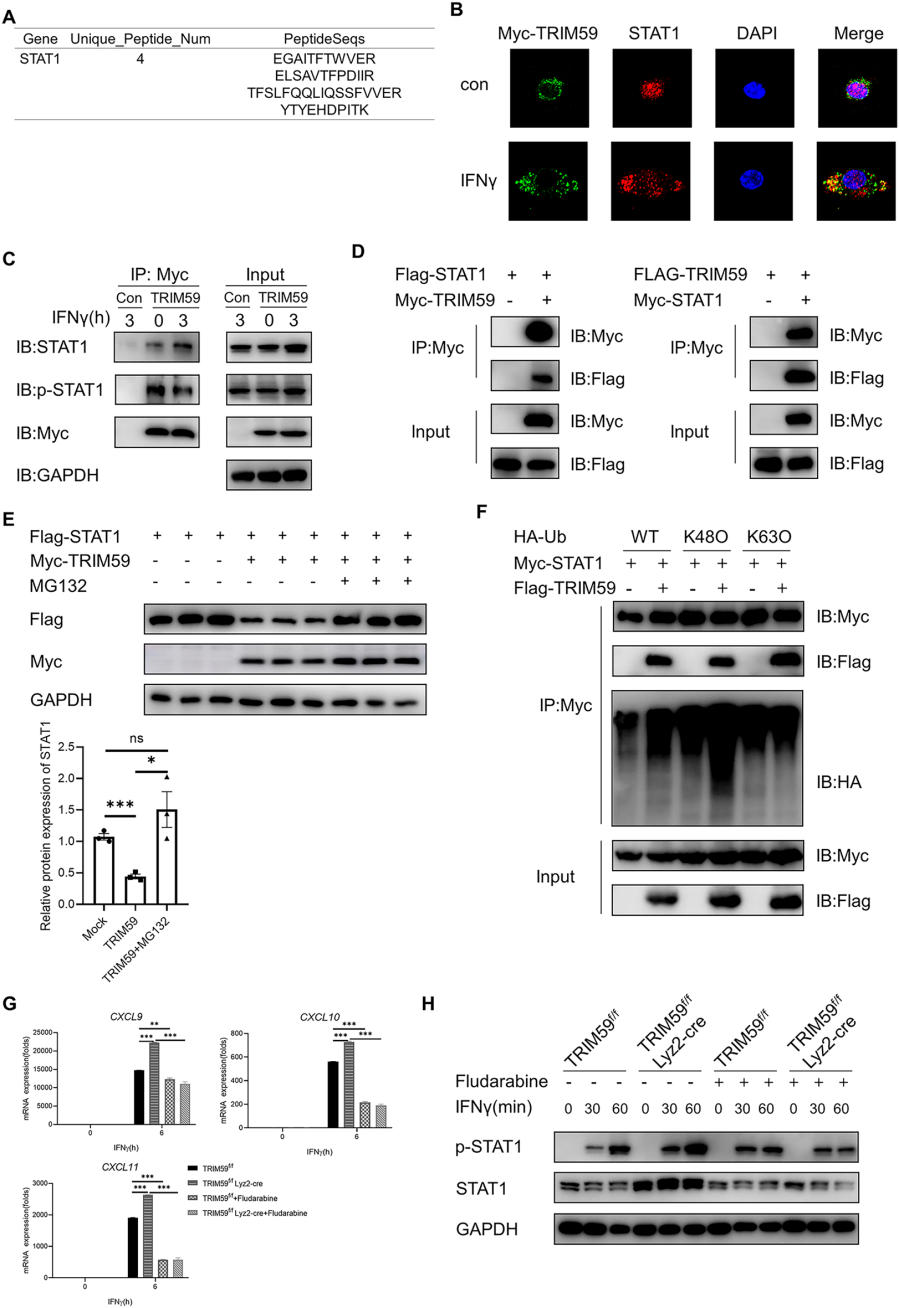
TRIM59 ubiquitinates and degrades STAT1. (**A**) The mass spectrometry results of the identification of protein. (**B**) The subcellular localization of TRIM59 (green) and STAT1 (red) in RAW264.7 cells were examined using confocal fluorescence microscopy. (**C**) The interaction between TRIM59 and STAT1 proteins was validated by Co-IP and IB analysis in TRIM59-overexpressing RAW264.7 cells. (**D**) The interaction between TRIM59 and STAT1 proteins was validated by Co-IP and IB analysis in HEK293T cells. (**E**) WB analysis of the protein level of STAT1 in HEK293T cells and quantitative analysis of STAT1 level normalized to GAPDH levels. (**F**) Myc-STAT1 and Flag-TRIM59 were co-transfected with HA-Ub, HA-Ub-K48O or HA-Ub-K63O. The ubiquitination of STAT1 was analyzed by Co-IP and WB analysis in HEK293T cells. (**G**) QPCR analysis of chemokines in BMDMs treated with IFNγ (40 ng/mL) for 6 h after pretreatment with fludarabine (10 μM, 1 h) (n = 3 per group). (**H**) WB analysis of STAT1 and p-STAT1 in BMDMs treated with IFNγ (40 ng/mL) for 30 and 60 min after pretreatment with fludarabine (10 μM, 1 h). The experiments were repeated three times. The data are represented as mean ± SEM, *p < 0.05, **p < 0.01, ***p < 0.001.

To elucidate the precise molecular mechanism underlying TRIM59-mediated activation M1 macrophage polarization, we conducted Myc-tagged TRIM59 co-immunoprecipitation (Co-IP) combined with mass spectrometry analysis. Among the identified proteins, STAT1, a key player in the IFNγ signaling pathway, garnered our attention (Fig. 5A). To validate mass spectrometry findings, we performed immunofluorescence staining and Co-IP experiments in TRIM59-overexpressing RAW264.7 cells. The results demonstrated that co-localization of TRIM59 with STAT1 upon IFNγ treatment (Fig. 5B). Additionally, TRIM59 was found to interact with STAT1 in TRIM59-overexpressing RAW264.7 cells (Fig. 5C). This interaction between TRIM59 and STAT1 was further confirmed through Co-IP assays conducted in HEK293T cells (Fig. 5D). Notably, the protein levels of STAT1 were reduced in HEK293T cells with overexpressing TRIM59, and this effect was reversed upon administration of MG132 (10 μM, 6 h) (Fig. 5E). This finding suggested that the STAT1 might be a substrate of TRIM59. Traditionally, proteasomal degradation has been associated with K48-linked ubiquitination. To investigate whether TRIM59 ubiquitinates STAT1 through K48-linked ubiquitination, we examined the levels of STAT1 ubiquitination in HEK293T cells co-transfected with Myc-STAT1 and Flag-TRIM59 with HA-Ub, HA-Ub-K48O or HA-Ub-K63O with MG132 treatment (10 μM, 6 h). The result demonstrated that TRIM59 facilitates K48-linked ubiquitination of STAT1 (Fig. 5F). To further verify that TRIM59 regulates macrophages polarization through the STAT1 signaling pathway, we used the inhibitor fludarabine. Fludarabine is a DNA synthesis inhibitor that specifically inhibits STAT1 and STAT1 phosphorylation, but has no effect on other STAT proteins. We pretreated BMDMs with Fludarabine (10 μM, 1 h), then stimulated them with IFNγ for 6 h. The results showed that the expression levels of the chemokines *CXCL9*, *CXCL10*, and *CXCL11* were significantly suppressed after fludarabine treatment in BMDMs (Fig. 5G). Meanwhile, we stimulated BMDMs with IFNγ after pretreatment with fludarabine (10 μM, 1 h) to detect the expression of STAT1 and p-STAT1. It was showed that fludarabine inhibited STAT1 and p-STAT1 protein levels in BMDMs (Fig. 5H). These findings provide compelling evidence that TRIM59 ubiquitinates and degrades STAT1.

### TRIM59 deficiency in macrophages inhibits tumor growth of MC38 by promoting M1 macrophages polarization

**Figure 6:**
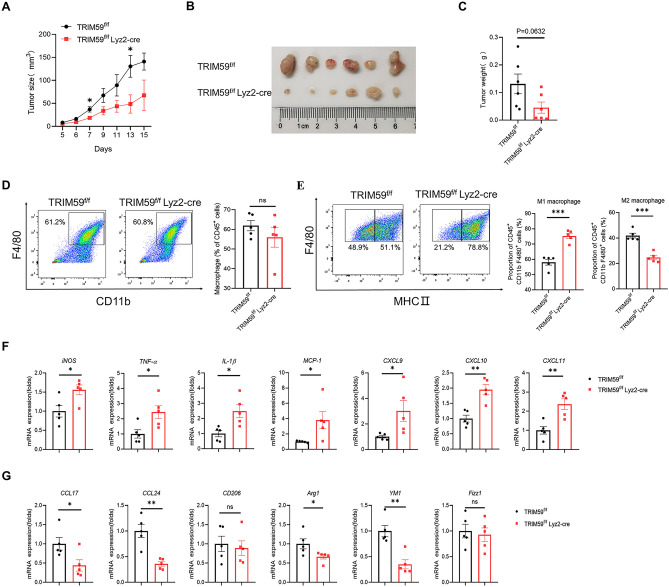
TRIM59 deficiency in macrophages inhibits tumor growth of MC38 by promoting M1 macrophages polarization. (**A**–**C**) Tumor growth curve (**A**), representative image of MC38 tumors (**B**), and tumor weight (**C**) in TRIM59^f/f^ and TRIM59^f/f^ Lyz2-cre mice (n = 6 per group). (**D**,**E**) Flow cytometry analysis of macrophage proportion (**D**), M1 macrophage and M2 macrophage proportion (**E**) (n = 5 per group). (**F**) QPCR analysis of chemokines and pro-inflammatory cytokines in MC38 tumors (n = 5 per group). (**G**) QPCR analysis of M2 marophage markers in MC38 tumors (n = 5 per group). The data are represented as mean ± SEM, *p < 0.05, **p < 0.01, ***p < 0.001.

Considering the impact of TRIM59 on macrophage activation, we proceeded to establish an additional mouse model. Specifically, we subcutaneously injected MC38 cells into both TRIM59^f/f^ and TRIM59^f/f^ Lyz2-cre mice. Interestingly, the tumor size exhibited a significant increase in TRIM59^f/f^ compared to TRIM59^f/f^ Lyz2-cre mice (Fig. 6A,B). Although the tumor weight did not reach statistical significance (*p* = 0.0632), there was a noticeable reduction in tumor burden in TRIM59^f/f^ Lyz2-cre mice (Fig. 6C). To explore changes in macrophage proportions, we performed flow cytometry analysis. The percentages of macrophages were not significantly different (Fig. 6D). However, in TRIM59^f/f^ Lyz2-cre mice, the proportion of M1 macrophages was upregulated while the proportion of M2 macrophages was downregulated (Fig. 6E). Several pro-inflammatory cytokines and chemokines were also detected in MC38 tumors. The levels of *iNOS*, *TNF-α*, *IL-1β*, *MCP-1*, *CXCL9*, *CXCL10*, and *CXCL11* were found to be higher in TRIM59^f/f^ Lyz2-cre mice (Fig. 6F). The M2 macrophage markers (*CCL17*, *CCL24*, *Arg1* and *YM1*) were reduced in TRIM59^f/f^ Lyz2-cre mice (Fig. 6G). These results indicate that TRIM59 deficiency in macrophages inhibits growth of colon carcinoma by promoting M1 macrophages polarization.

## Discussion

In this study, we generated TRIM59 conditional knockout mice to investigate the impact of this protein on the occurrence and development of CRC by regulating macrophages polarization. We found that TRIM59 deficiency suppressed colorectal tumorigenesis by promoting M1 macrophage activation through the STAT1 signaling pathway. Moreover, as an E3 ligase, TRIM59 interacted with STAT1 and facilitated its degradation.

Previous studies have demonstrated the role of TRIM59 in macrophages. Zhu et al. conducted a study that revealed the promotion of phagocytosis activity by TRIM59^40^, as well as its protective effects against sepsis by regulating inflammation and phagocytosis in macrophages^41^. This research demonstrated an increase in the level of *TNF-α*, *iNOS*, *IL-6*, and *IL-1β* in TRIM59-deficient BMDMs following LPS stimulation^41^. Furthermore, Yang et al. found that silencing TRIM59 in RAW264.7 cells resulted in the upregulation of *TNF-α*, *iNOS*, *IL-6*, and *IL-1β* after LPS treatment^46^. However, our study revealed that TRIM59 deficiency had a minimal effect on the signaling pathways induced by LPS, including MAPK and NF-κB pathways. The induction of pro-inflammatory cytokines by LPS in TRIM59-deficient BMDMs was limited, resulting in only minor alterations. Moreover, TRIM59 deficiency in macrophages did not affect DSS-induced colitis or LPS-induced sepsis in mice. We believe that TRIM59 is more likely to be involved in chronic inflammation rather than acute inflammation. Following IFNγ stimulation in BMDMs, we found that TRIM59 participated in the STAT1 signaling pathway. The protein level of STAT1 and STAT1 phosphorylation were upregulated in TRIM59-deficient BMDMs, subsequently regulating the expression of downstream genes, especially the chemokines *CXCL9*, *CXCL10*, and *CXCL11*. These chemokines play a crucial role in macrophage-mediated tumor immunity by facilitating the recruitment of other immune cells within the TME^47,48^. Thus, our results demonstrate that TRIM59 deficiency promotes M1 macrophage activation via the STAT1 signaling pathway.

Although previously studies have reported the function of TRIM59 in macrophages in tumors, there have been inconsistent and conflicting conclusions. Zhu et al. found that the loss of TRIM59 promoted melanoma migration and invasion in transplanted mice with myeloid-specific deletion of TRIM59 by upregulating MMP-9 and Madcam1^49^. In contrast, Yang et al. found that TRIM59-overexpression in mouse bone marrow cells promoted melanoma growth^46^. Additionally, Geng et al. provided evidence that TRIM59 stimulated macrophages to facilitate lung cancer growth and metastasis using a CD11b promoter-driven macrophage-specific TRIM59 transgenic mouse model^50^. In our study, we have provided evidence supporting the inhibitory effect of TRIM59 deficiency in macrophages on CRC, including CAC and MC38 transplanted CRC models, which is consistent with the findings of Yang et al.^46^ and Geng et al.^50^. It is important to acknowledge that cancer is heterogeneous and dynamic. The same gene may play different roles in different tumors, as tumors have distinct progression processes. Therefore, it is crucial to recognize that tumor characteristics and the TME may vary across different cancer types and stages^51^. Consequently, further research is needed to elucidate the specific mechanisms by which TRIM59 regulates macrophages in different types of cancer.

TRIM59, a member of the TRIM family, has been identified to possess an E3 ubiquitin ligase function. Previous studies have demonstrated the ubiquitination function of TRIM59 in various contexts. For instance, TRIM59 has been shown to promote the degradation of p53 in gastric tumors^32^, regulate the cell cycle by degrading protein phosphatase 1B in hepatocellular carcinoma^34^, and promote macroH2A1 ubiquitination in glioblastoma^52^. In our study, we employed mass spectrometry and identified STAT1 as a substrate of TRIM59. STAT1 is a crucial transcription factor involved in the regulation of macrophages, particularly their differentiation into M1 macrophages^53^. Upon IFNγ stimulation, the JAK-STAT1 signaling pathway is activated, resulting in the phosphorylation of STAT1 and subsequent polarization of M1 macrophages^54^. Our study confirmed the interaction between TRIM59 and STAT1 through Co-IP and immunofluorescence assays, which aligns with previous research^46^. However, we provided a novel explanation for the underlying mechanism. We observed that TRIM59 facilitated the degradation of STAT1 through K48-linked ubiquitination in HEK293T cells, whereas TRIM59 deficiency in BMDMs led to an upregulation of STAT1 expression. The STAT1 inhibitor fludarabine inhibited the expression of STAT1 and p-STAT1 in TRIM59-deficient BMDMs treated with IFNγ, as well as the chemokines *CXCL9*, *CXCL10* and *CXCL11*. These findings suggest that TRIM59 regulates M1 macrophage activation through the STAT1 signaling pathway. Nevertheless, further investigation is required to full eludcidate the specific mechanism by which TRIM59 mediates STAT1 degradation.

In summary, our study has successfully demonstrated that TRIM59 deficiency in macrophages can effectively promote M1 macrophage activation, thereby suppressing colorectal tumorigenesis. Additionally, our findings have revealed the interaction between TRIM59 and STAT1, where TRIM59 acts as a ubiquitin ligase to target STAT1 for degradation. These results strongly suggest that the TRIM59/STAT1 axis holds significant promise as a novel immunotherapeutic strategy for the treatment of colon carcinoma.

### Supplementary Information

Supplementary Information 1.

Supplementary Information 2.

## Data Availability

All necessary data are included in paper. The remaining data can be provided by corresponding authors on reasonable request.
