# Supplementary material for: TRIM59 deficiency promotes M1 macrophage activation and inhibits colorectal cancer through the STAT1 signaling pathway
Source: Sci Rep. 2024 Jul 12;14:16081. doi: 10.1038/s41598-024-66388-0 (PMC11239810; doi:10.1038/s41598-024-66388-0)

**Supplementary data**

**TRIM59 deficiency promotes M1 macrophage activation and inhibits colorectal cancer through the STAT1 signaling pathway**

Haidong Wang^1#^, Jun Lou^1#^, Hao Liu^2^, Yunlong Liu^1^, Binbin Xie^1^,Wei Zhang^1^, Jiansheng Xie^2^, Hongming Pan^1*^, Weidong Han^1,3*^

^1^Department of Medical Oncology, Sir Run Run Shaw Hospital, School of Medicine, Zhejiang University, Hangzhou, Zhejiang, China; ^2^Laboratory of Cancer Biology, Institute of Clinical Science, Sir Run Run Shaw Hospital, School of Medicine, Zhejiang University, Hangzhou, Zhejiang, China; ^3^Department of Colorectal Medical Oncology, Zhejiang Cancer Hospital, No. 1, East Banshan Road, Gongshu District, Hangzhou , P.R. China 310022.

#Equal contributors

*Corresponding Authors: Weidong Han, Sir Run Run Shaw Hospital, School of Medicine, Zhejiang University, 3# East Qingchun Road, Hangzhou, Zhejiang, China, 310016. Phone: +86-571-86006926; E-mail: hanwd@zju.edu.cn; Hongming Pan, Sir Run Run Shaw Hospital, School of Medicine, Zhejiang University, 3# East Qingchun Road, Hangzhou, Zhejiang, China, 310016. Phone: +86-571-86006926; E-mail: panhongming@zju.edu.cn.

Supplementary Figures 1-6

Supplementary Table S1-2

Original western blots for Figure 3., Figure 4., Figure 5., Supplementary Figure 2., Supplementary Figure 3. and Supplementary Figure 5**.**

**Supplementary Figures**

**
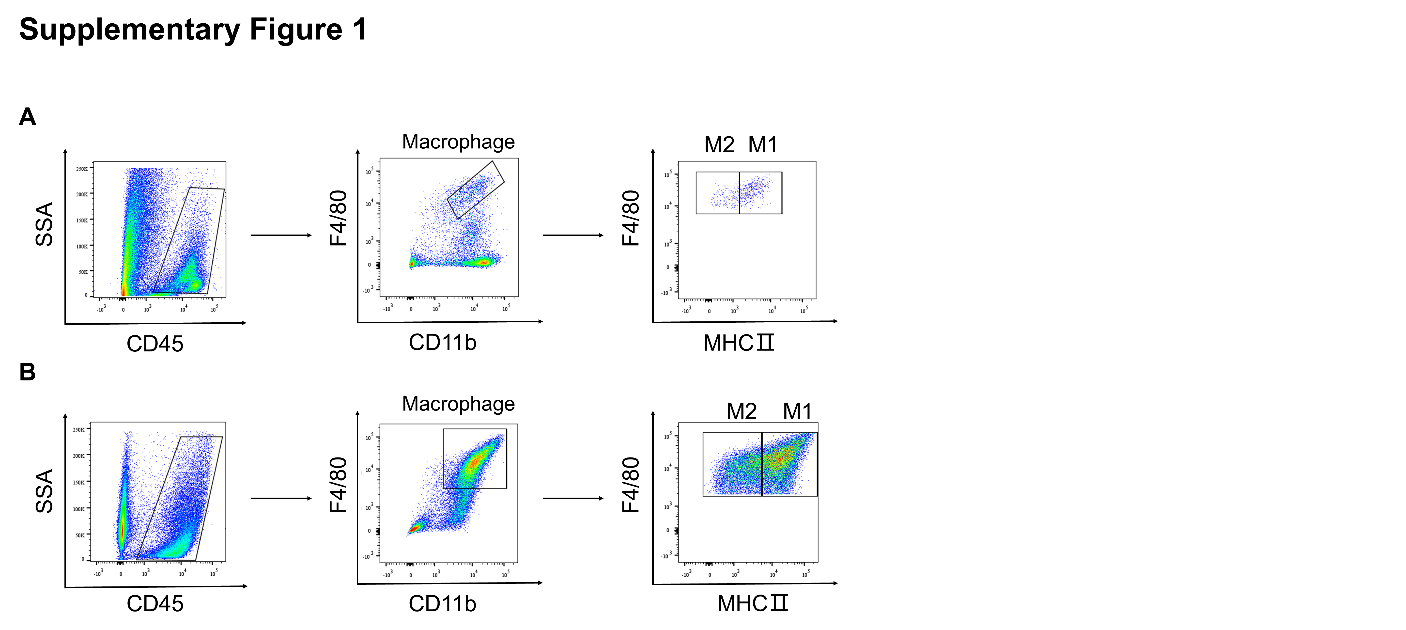
**

**Supplementary Figure 1.** Flow cytometry analysis process of macrophage. (A) The analysis process of macrophage in CAC tumors. (B) The analysis process of macrophage in MC38 tumors.

**
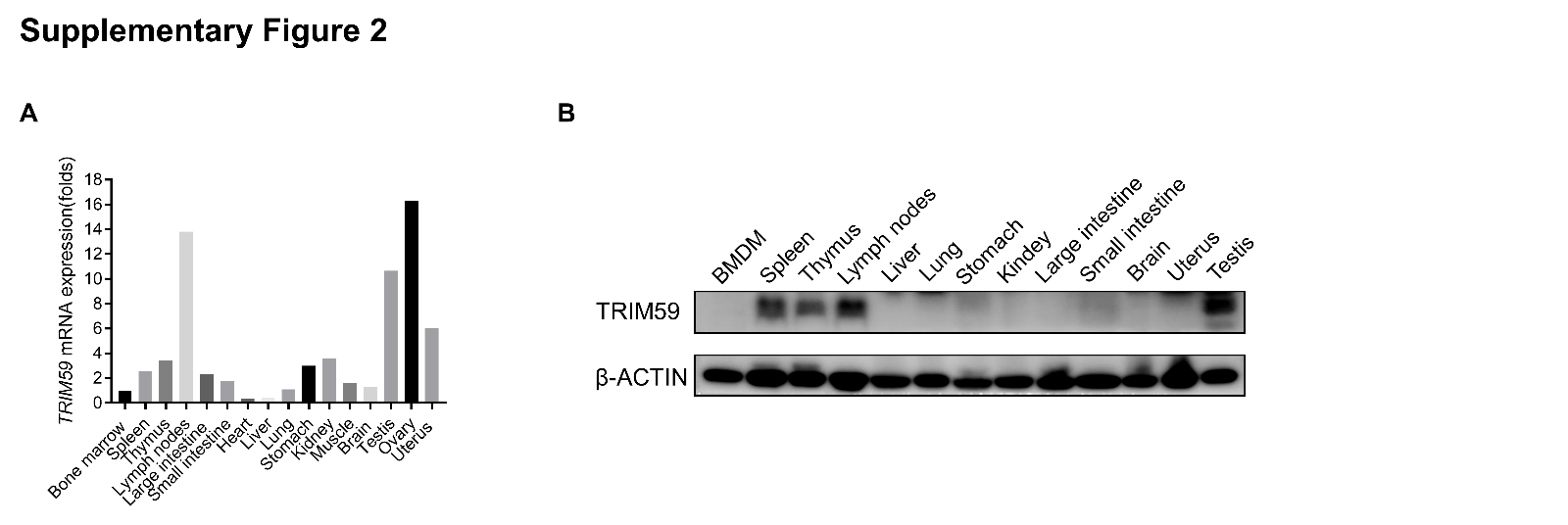
**

**Supplementary Figure 2.** The expression of TRIM59 in different mouse tissues. (A- B) The mRNA level (A) and the protein expression (B) of TRIM59 in different mouse tissues.

**
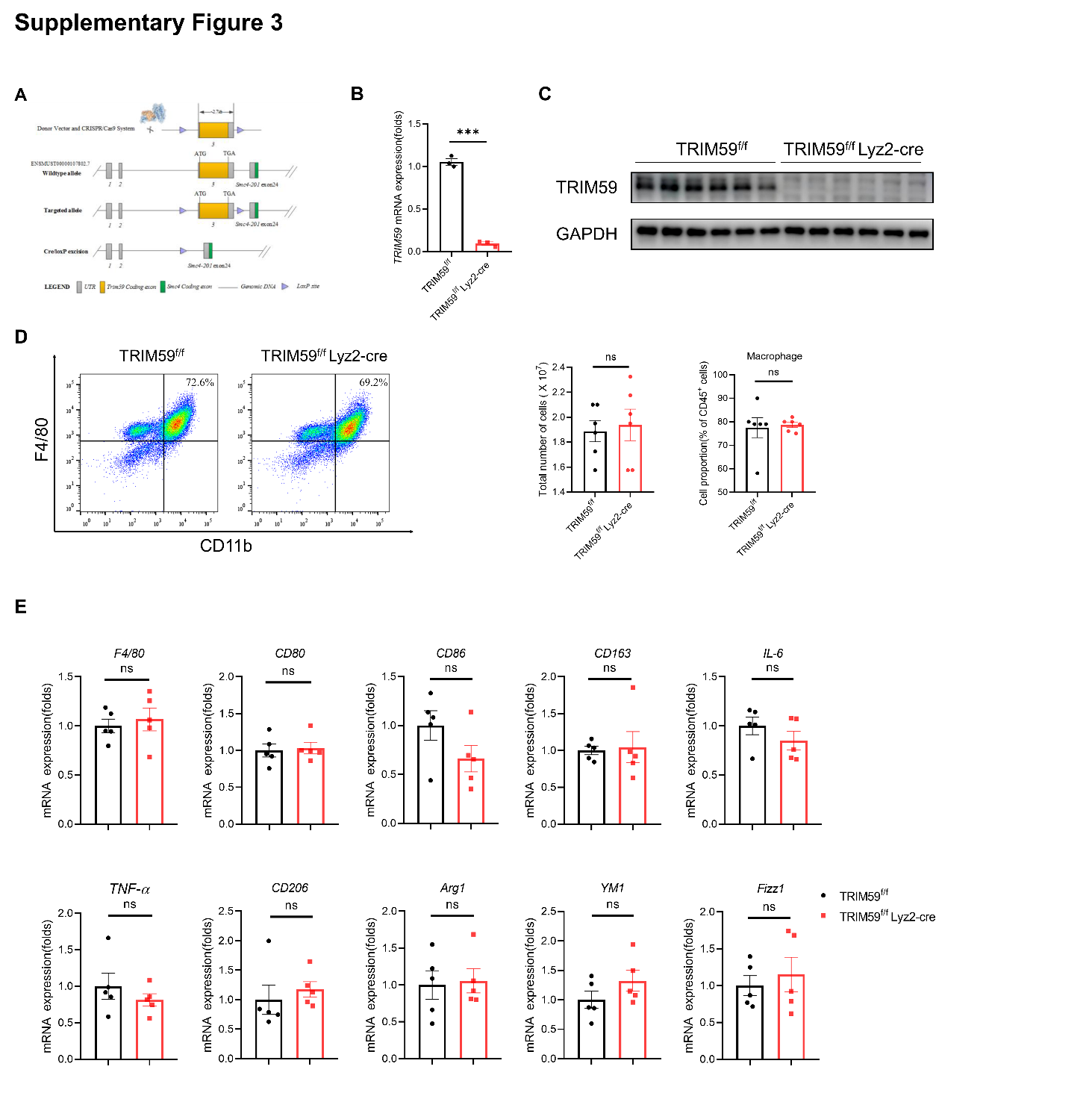
**

**Supplementary Figure 3.** TRIM59 deficiency in macrophages does not affect macrophage maturation. (A) The schematic diagram of TRIM59 conditional knockout mouse construction. (B, C) QPCR (B) and WB (C) analysis of TRIM59 expression in BMDMs. (D) Flow cytometry analysis of macrophage in BMDMs (n = 6 per group). (E) QPCR analysis of macrophage surface markers in BMDMs (n = 5 per group). The data are represented as mean ± SEM, *p < 0.05, **p < 0.01, ***p <0.001.

**
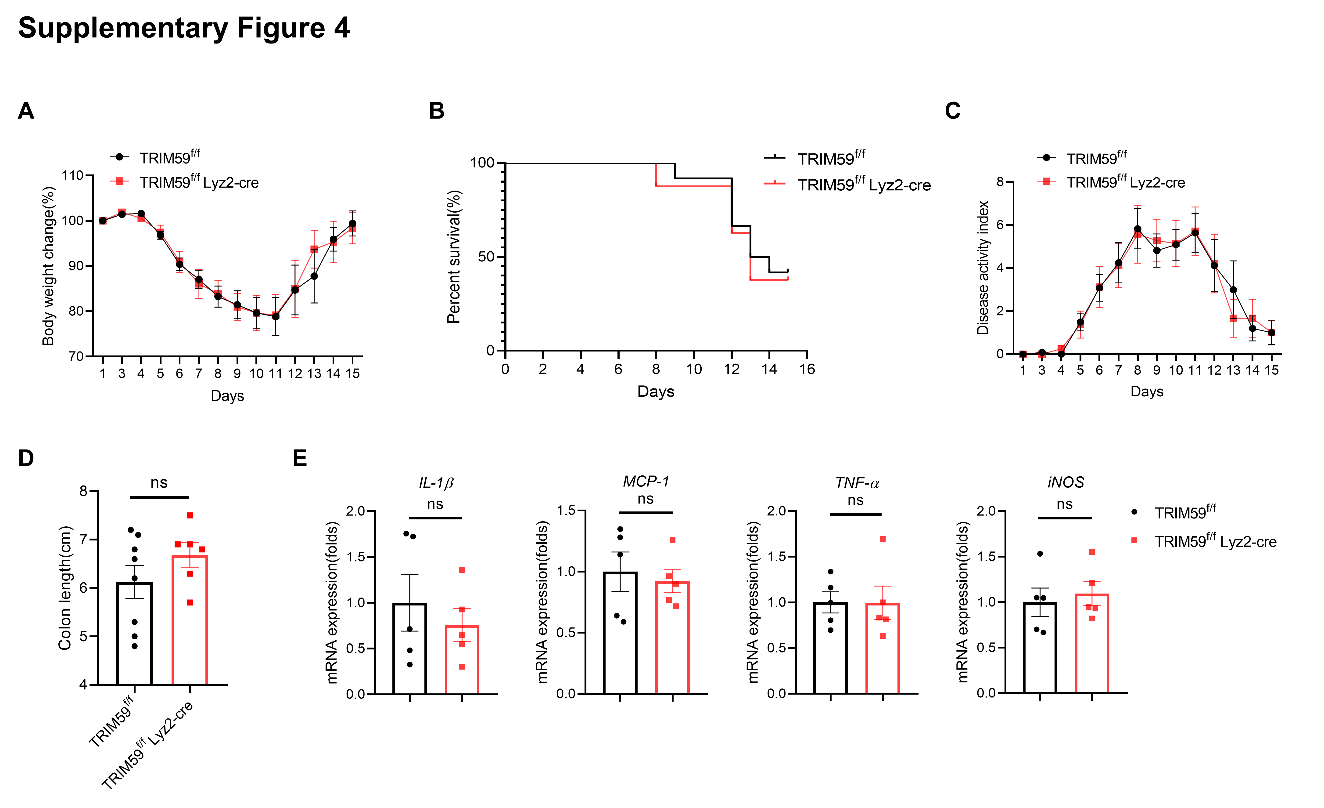
**

**Supplementary Figure 4.** TRIM59 deficiency in macrophages does not affect DSS-induced colitis. (A- D) The percentage change in body weight (A), survival curve (B) and the disease activity index (C) of colitis mice (n = 8-12 per group). (D) The colon length of colitis mice (n = 6-8 per group). (E) QPCR analysis of pro-inflammatory cytokines in the colorectum of colitis mice (n = 5 per group). The data are represented as mean ± SEM, *p < 0.05, **p < 0.01, ***p <0.001.

**
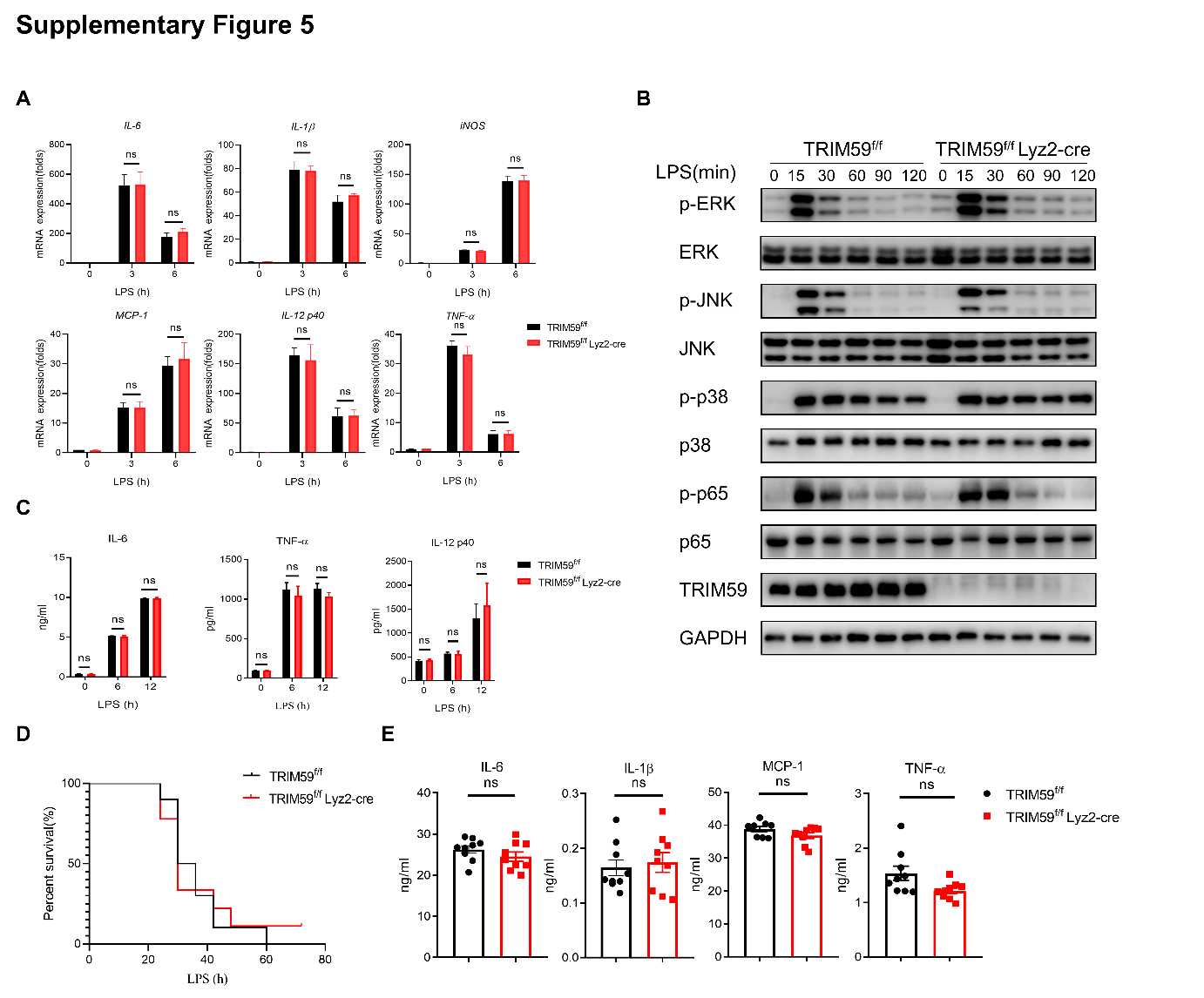
**

**Supplementary Figure 5.** TRIM59 deficiency in macrophages does not affect LPS-induced sepsis in mice. (A) QPCR analysis of pro-inflammatory cytokines in BMDMs treated with LPS (500 ng/mL) for 3 and 6 h (n = 3 per group). (B) WB analysis of NF-κB and MAPK signaling pathways in BMDMs treated with LPS (500 ng/mL) for 0, 15, 30, 60, 90 and 120 min. (C) ELISA analysis of pro-inflammatory cytokines in the supernatant of BMDMs treated with LPS (500 ng/mL) for 6 and 12 h (n = 3 per group). (D) The survival curve of the sepsis mice model (n = 9-10 per group). (E) ELISA analysis of pro-inflammatory cytokines in the serum of sepsis mice (n = 9 per group). The experiments were repeated three times. The data are represented as mean ± SEM, *p < 0.05, **p < 0.01, ***p <0.001.

**Supplemental Tables**

**Table S1**

QPCR mouse primer sequences

| genes | sequences |
| --- | --- |
| β-ACTIN | F: AGAGGGAAATCGTGCGTGAC  R: CAATAGTGATGACCTGGCCGT |
| TRIM59 | F: GCCTCGCGAATAACAAAGGA  R: GCTGTAACAGATGGGACACG |
| IL-1β | F：GCTGAAAGCTCTCCACCTCA  R：AGGCCACAGGTATTTTGTCG |
| IL-6 | F：GAGGATACCACTCCCAACAGACC  R：AAGTGCATCATCGTTGTTCATACA |
| IL-10 | F: GGTTGCCAAGCCTTATCGGA  R: ACCTGCTCCACTGCCTTGCT |
| IL-12 p40 | F: GGAAGCACGGCAGCAGAATA  R: AACTTGAGGGAGAAGTAGGAATGG |
| TNF-α | F: AGTGACAAGCCTGTAGCCC  R: GAGGTTGACTTTCTCCTGGTAT |
| iNOS | F：AACGGAGAACGTTGGATTTG  R：CAGCACAAGGGGTTTTCTTC |
| MCP-1 | F: CTTCTGGGCCTGCTGTTCA  R: CCAGCCTACTCATTGGGATCA |
| Arg1 | F: TGGCTTGCGAGACGTAGAC  R: GCTCAGGTGAATCGGCCTTTT |
| CD206 | F: CTTCATCTTCGGGCCTTTGG  R: TGACCACTCCTGCTGCTTTA |
| YM1 | F: ATGAAGGAGCCACTGAGGTC  R: CCACGGCACCTCCTAAATTG |
| Fizz1 | F: AGCTGATGGTCCCAGTGAAT  R: ACACCCAGTAGCAGTCATCC |
| F4/80 | F: TTTCCTCGCCTGCTTCTTC  R: CCCCGTCTCTGTATTCAACC |
| CD80 | F: CCCCAGAAGACCCTCCTGATAG  R: CCGAAGGTAAGGCTGTTGTTTG |
| CD86 | F: ACGTATTGGAAGGAGATTACAGCT  R: TCTGTCAGCGTTACTATCCCGC |
| CD163 | F: GGCTAGACGAAGTCATCTGCAC  R: CTTCGTTGGTCAGCCTCAGAGA |
| CXCL9 | F:AAAATTTCATCACGCCCTTG  R:TCTCCAGCTTGGTGAGGTCT |
| CXCL10 | F: GCCGTCATTTTCTGCCTCA  R: CGTCCTTGCGAGAGGGATC |
| CXCL11 | F:GGCTTCCTTATGTTCAAACAGGG  R:GCCGTTACTCGGGTAAATTACA |
| STAT1 | F:GACCACCTCTCTTCCTGTCG  R:TGCCAACTCAACACCTCTGA |
| IRF1 | F:CCTGGGTCAGGACTTGGATA  R: TTCGGCTATCTTCCCTTCCT |
| IRF8 | F:AGCTGGGTTACATGCTCCAC  R:TAGCACTCCTTGAGGCAGGT |

**Table S2**

Antibody used in this study

| Antibody | Cat.No | Supplier | Dilution | Application |
| --- | --- | --- | --- | --- |
| β-ACTIN | R1207-1 | Huabio | 1: 2000 | WB^†^ |
| GAPDH | EM101 | Huabio | 1: 2000 | WB |
| TRIM59 | 28575-1-AP | Proteintech | 1: 1000 | WB |
| Flag | 14793 | CST | 1: 1000 | WB |
| Myc | 2276 | CST | 1: 1000 | WB/IF^‡^ |
| HA | 3724 | CST | 1: 1000 | WB |
| JAK1 | 3344 | CST | 1: 1000 | WB |
| p-JAK1 | 3331 | CST | 1: 1000 | WB |
| JAK2 | 3230 | CST | 1: 1000 | WB |
| p-JAK2 | 3771 | CST | 1: 1000 | WB |
| STAT1 | 14994 | CST | 1: 1000 | WB |
| p-STAT1 | 7649 | CST | 1: 1000 | WB |
| p38 | 8690 | CST | 1: 1000 | WB |
| p-p38 | 4551 | CST | 1: 1000 | WB |
| p65 | 8242 | CST | 1: 1000 | WB |
| p-p65 | 3033 | CST | 1: 1000 | WB |
| ERK | 4695 | CST | 1: 1000 | WB |
| p-ERK | 4370 | CST | 1: 1000 | WB |
| JNK | 9252 | CST | 1: 1000 | WB |
| p-JNK | 4668 | CST | 1: 1000 | WB |
| Myc | 2276 | CST | 1: 250 | IP^§^ |
| STAT1 | 14994 | CST | 1: 200 | IF |

^†^WB: Western blotting. ^‡^IF: Immunofluorescence. ^§^IP: Immunoprecipitation.

Original western blots


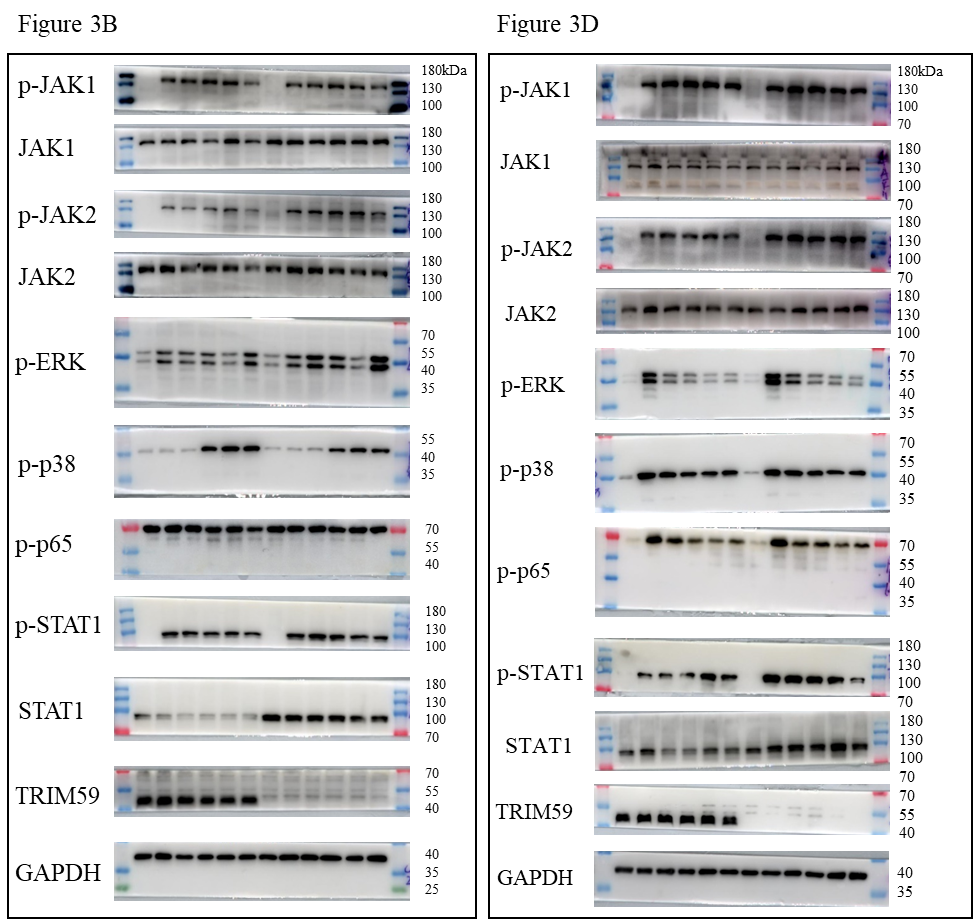


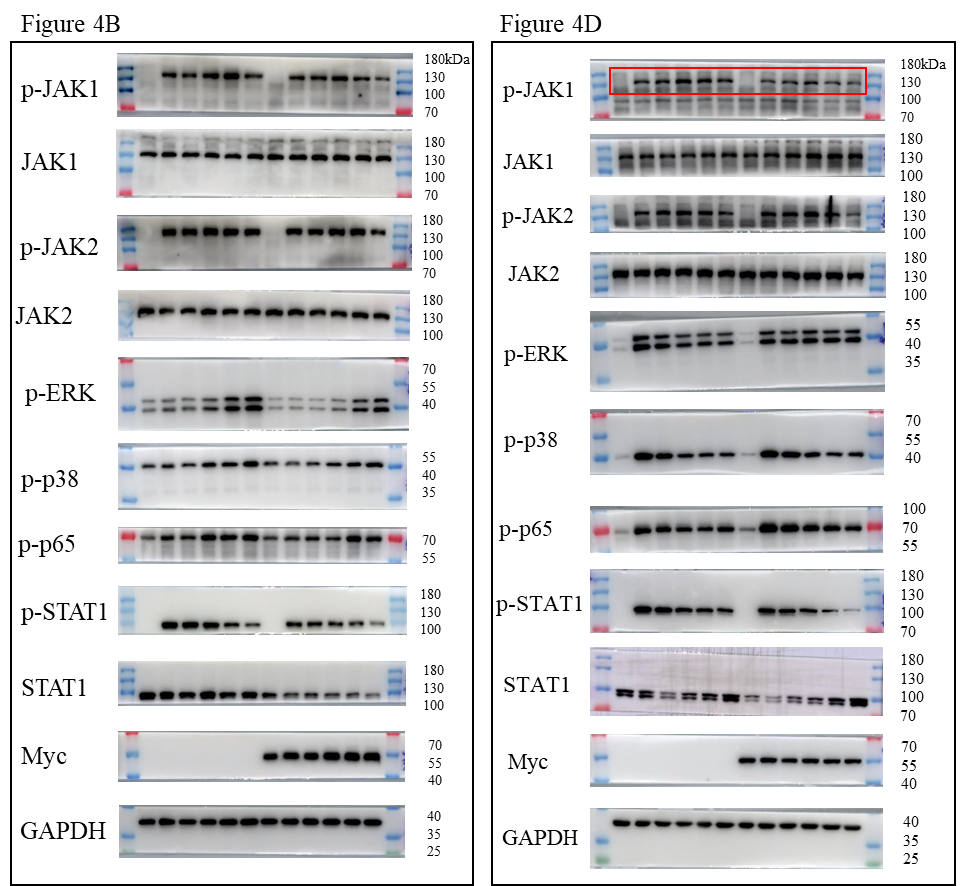


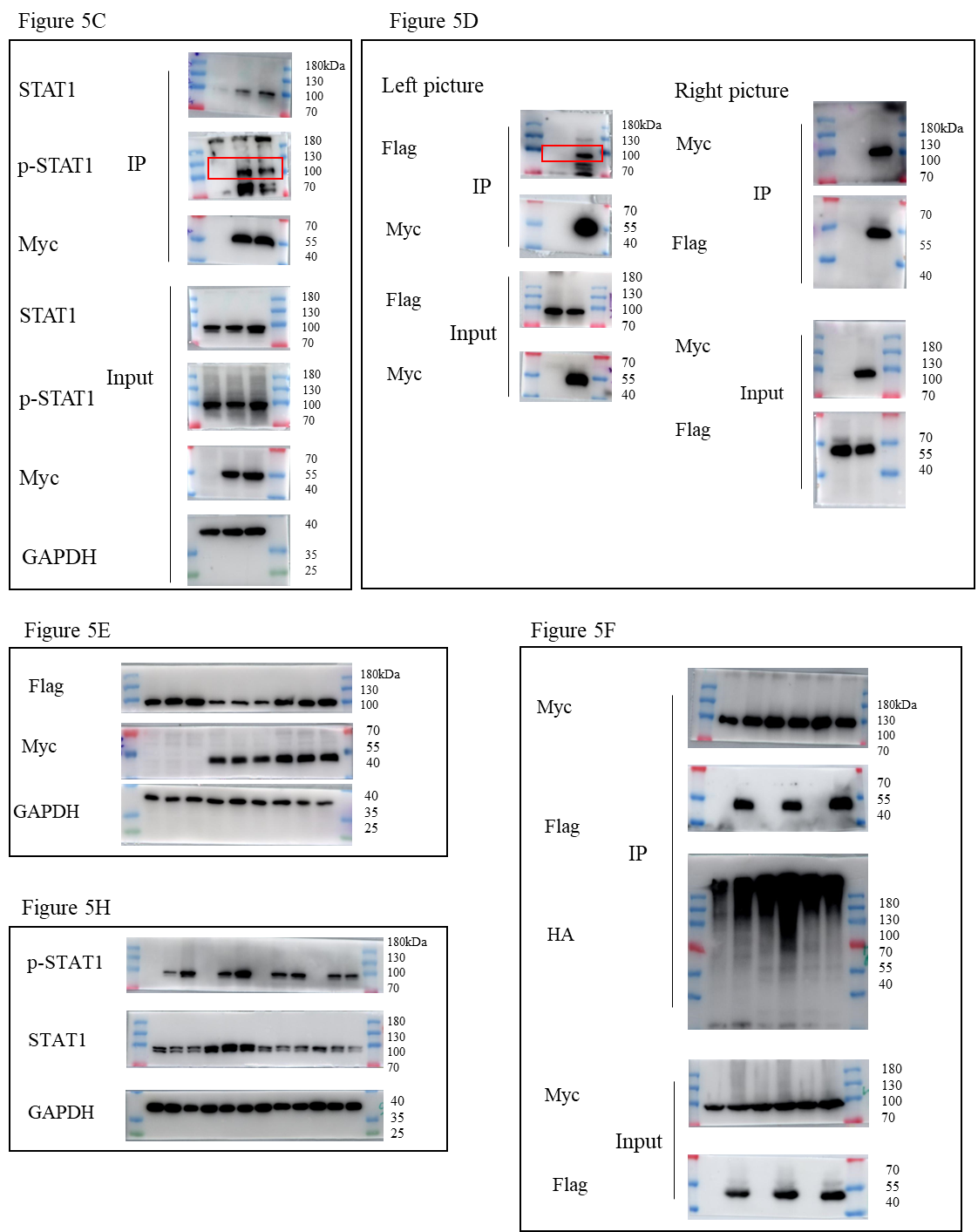


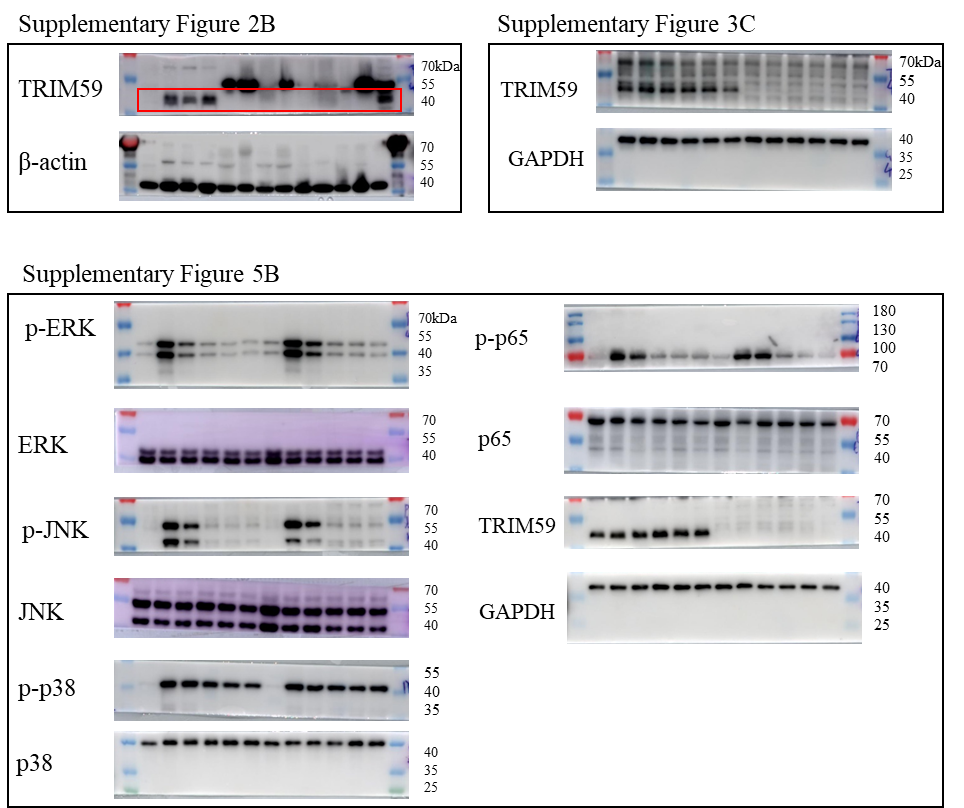

Supplement: Supplementary file 2 — Supplementary Information 2. [file 41598_2024_66388_MOESM2_ESM.docx]
